# Challenges and Promises of PET Radiomics

**DOI:** 10.1016/j.ijrobp.2017.12.268

**Published:** 2018-11-15

**Authors:** Gary J.R. Cook, Gurdip Azad, Kasia Owczarczyk, Musib Siddique, Vicky Goh

**Affiliations:** Department of Cancer Imaging, School of Biomedical Engineering and Imaging Sciences, King's College London, London, United Kingdom

## Abstract

**Purpose:**

Radiomics describes the extraction of multiple, otherwise invisible, features from medical images that, with bioinformatic approaches, can be used to provide additional information that can predict underlying tumor biology and behavior.

**Methods and Materials:**

Radiomic signatures can be used alone or with other patient-specific data to improve tumor phenotyping, treatment response prediction, and prognosis, noninvasively. The data describing ^18^F-fluorodeoxyglucose positron emission tomography radiomics, often using texture or heterogeneity parameters, are increasing rapidly.

**Results:**

In relation to radiation therapy practice, early data have reported the use of radiomic approaches to better define tumor volumes and predict radiation toxicity and treatment response.

**Conclusions:**

Although at an early stage of development, with many technical challenges remaining and a need for standardization, promise nevertheless exists that PET radiomics will contribute to personalized medicine, especially with the availability of increased computing power and the development of machine-learning approaches for imaging.

**Summary**

Radiomics describes the extraction of multiple features from medical images using bioinformatic approaches to provide additional information to predict underlying tumor biology and behavior. In relation to PET radiomics in radiation therapy practice, early data have reported radiomic approaches to define tumor volumes and predict radiation toxicity and treatment response. With many technical challenges remaining and a need for standardization, promise nevertheless exists that PET radiomics will contribute to personalized medicine.

## Introduction

Radiomics is a relatively new and evolving field in medical imaging in which a large number of features are extracted from medical images for analysis and interpretation using bioinformatic approaches 1, 2, 3, 4. It is assumed that medical images contain more information than can be appreciated by eye, and the underlying hypothesis on which radiomics relies is that a relationship exists between such extracted image parameters and the tumor molecular phenotype and/or genotype. The study of radiomics is increasing and has become of greater academic interest since it has been recognized that genetic heterogeneity exists within tumors and between metastatic tumors in the same patient. Genotypic heterogeneity contributes to the development of subpopulations of cells with divergent biological behavior that are resistant to treatment (5). At the biological level, it has been recognized that heterogeneity of the tumor microenvironment might be reflected in medical images, with respect to cellular density, proliferation, angiogenesis, hypoxia, receptor expression, necrosis, fibrosis, and inflammation and that these factors can contribute to poor treatment responses and a more aggressive phenotype (6).

Therefore, interest is increasing in using radiomic signatures to better determine the tumor phenotype. This would allow for whole tumor segmentation or segmentation of tumor subregions with different biological characteristics that might contribute to treatment resistance and better prediction and evaluation of the treatment response and prognostication. Radiomic features can be used alone or combined with other clinical or -omics data (eg, radiogenomics) 7, 8.

In addition to standard or semantic features, such as tumor dimension and volume or density/signal intensity on computed tomography (CT) and magnetic resonance imaging (MRI) or the standardized uptake value (SUV) with positron emission tomography (PET), many other parameters can be extracted from images. These relate to the voxel-intensity volume histogram, spatial heterogeneity between voxels of different intensities (texture analysis), and tumor shape and surface outline, among other factors, with the possibility of obtaining hundreds of features from a single tumor 9, 10, 11.

The attractive characteristics of radiomics are the ability to sample the whole tumor, negating the sampling error that occurs with tissue biopsies and that the data can be extracted noninvasively and using serial examinations. Most radiomic features can be extracted from images that have been acquired using standard image protocols and thereby offer “free” additional information with only the need for postprocessing. The disadvantages of the radiomics approach include that the extracted image parameters relate to the relatively macroscopic scale and are therefore unlikely to bear a direct relationship to underlying cellular biology on a microscopic scale. Also, image parameters derived from mathematical formulas might, in themselves, not correspond to visual perception. For example, a preclinical study of an orthotopic breast cancer model reported that although some texture features are sensitive to the spatial distribution of cells and others to the cellular density, they are not easily correlated to the histologic texture or able to capture tumor cell heterogeneity (12).

Spatial resolution and, hence, voxel dimensions vary between and within the different clinical imaging modalities used in oncology from submillimeter with CT and MRI to several millimeters with PET and single photon emission CT. In addition to the larger voxels and resultant coarser tumor sampling, PET and single photon emission CT also show a further disadvantage with an inherently lower signal/noise ratio. It is also unlikely that the features extracted from a particular imaging modality will have the same association with the underlying tumor characteristics as those from another imaging modality. For example, CT measures the tissue density inferred from absorption of X rays but ^18^F-fluorodeoxyglucose (^18^F-FDG) PET measures cellular glucose metabolism, and MRI measures differences in proton density and relaxation properties. These differences pose both challenges and opportunities for hybrid imaging (eg, PET/CT or PET/MRI), such that registered images might provide incremental or complementary information compared with a single image data set. Although most of the PET radiomics data have described ^18^F-FDG, examples have also been reported of the use of radiomics to predict the chemotherapy response in breast cancer using ^18^F-fluorothymidine (13) or for grading and prognosis in high-grade gliomas using ^18^F-fluoroethyltyrosine (14).

Several technical challenges remain in the evaluation and implementation of radiomic approaches in the clinic, and we have discussed some of these further, together with some of the early reported findings of PET radiomics that relate to radiation therapy practice and might eventually translate into the clinic.

## Technical Aspects

Many of the additional parameters used in PET radiomics relate to intratumoral heterogeneity 15, 16, 17, although other features that relate to shape or other metrics have also been described 18, 19. In the use of machine-learning algorithms, such as with convolutional neural networks, the features that combine to provide the radiomic signature might be unknown (20). However, knowledge of spatial heterogeneity in tumors is of interest in its own right, in addition to being a part of selecting radiomic signatures. The most commonly used methods include statistical features (first-, second-, high-order). However, other methods include model-based features (eg, fractals) or transform-based approaches, which convert the spatial information in an image into frequency (Fourier) information or scale and frequency (wavelet) information 9, 21, 22.

First-order features represent global measurements of a tumor that do not convey any spatial information. Standard parameters such as SUV, metabolic volume (MV), and total lesion glycolysis (the product of the mean SUV and MV) fall within this group. However, other features that can be derived from a histogram of voxel intensity frequencies describe global heterogeneity (eg, skewness [asymmetry] and kurtosis [peakedness], describing the shape of the histogram) and entropy and energy (also named uniformity), describing the randomness and homogeneity of voxel values, respectively. Second- and high-order statistical features contain information on spatial relationships between the intensities of ≥2 voxels and are derived from co-occurrence or difference matrices to give local or regional measures of heterogeneity, often called “texture analysis” (10). Also, run length and size zone matrices give information on runs of voxels or groups of similar voxels in a certain direction (23). Commonly used second-order features include entropy, energy, homogeneity, and contrast (which should not be confused with first- or high-order features that bear the same names), which measure the relationships between pairs of voxels. High-order features include those derived from neighborhood gray-tone difference matrices, such as coarseness, contrast, busyness, and complexity, which describe the relationships between a voxel and those in neighboring planes. These features are thought to best represent the human perception of heterogeneity or texture within an image (11).

Many hundreds of features have been described and can be extracted from a tumor volume of interest, whatever the imaging modality, and this in itself can pose challenges to avoid overfitting and being able to manage collinearity and, hence, the redundancy of features. The more features tested in a model, the more samples that are required. Thus, unless a small number of features that have previously shown predictive value and robustness are to be used in a hypothesis-driven approach, the data from hundreds of patients might be required to avoid false-positive associations. Large samples are especially required for machine-learning methods in which hundreds or thousands of data sets might be required to train the algorithm when no a priori assumptions are available regarding the meaning or strength of the individual features.

Although PET scans performed for clinical purposes can be retrospectively postprocessed for feature extraction, this can present a further challenge in mining larger shared data sets because interinstitutional (or even intrainstitutional) standardization of image acquisition with PET is likely to be lacking. This results from the variabilities in scanner hardware from different manufacturers, injected activity, acquisition time after injection, acquisition time per bed position, CT parameters used for attenuation correction of the PET data, matrix size, and slice thickness. For example, it has been shown that the calculated texture features can vary depending on the time an ^18^F-FDG PET/CT scan is acquired after injection (24) and are also dependent on the voxel dimensions 25, 26.

An additional factor in PET scan acquisition that has been shown to affect measurement of texture features is respiratory motion, because PET acquisitions typically require a few minutes for each bed position. Respiratory gating produces different, but probably more accurate, results because blurring due to motion is minimized but is not routine in clinical acquisitions 27, 28.

Good progress has been made in standardizing ^18^F-FDG PET/CT protocols in oncologic imaging in the United States and Europe 29, 30, and a number of recommendations are available on how to develop new imaging biomarkers 31, 32, 33. In particular, some recommendations on how the current limitations on the use of texture analysis should be addressed have recently been reported (17). However, as yet, the field of PET radiomics is far from standardized, making it difficult to pool data or perform meta-analyses.

Other factors that can vary between institutions and might influence measurement of texture features include the reconstruction algorithm adopted and the presence of postreconstruction smoothing, which is commonly applied to clinical images. Some features, such as first-order entropy, appear to be relatively robust with little variation using different reconstruction parameters. However, 40 of 50 other features were reported to show >30% variation when calculated after the use of 5 different reconstruction parameters in a previous study (34).

The aspect of the radiomic workflow that probably causes the most controversy is the choice of the segmentation method to delineate a tumor, for which a compromise is often required between accuracy and reproducibility. The method varies between manually drawn regions of interest, which are subject to the greatest inter- and intraobserver variability, to automatic or semiautomatic methods, such as using a fixed percentage threshold of the maximum SUV (SUVmax), commonly 40%, to more sophisticated methods such as fuzzy locally adaptive Bayesian (35). A fixed threshold method can underestimate the true tumor volume by ignoring areas of low activity but is highly reproducible. In contrast, the fuzzy locally adaptive Bayesian method is better suited for heterogeneous volumes with low resolution and variable noise and contrast, such as is typical with PET, and has been reported to be accurate and reproducible in phantom and patient data 36, 37. Although necrotic tissue without ^18^F-FDG uptake is a relatively common finding, it remains unclear whether necrotic subregions should be included when segmenting tumors for texture analysis. It also remains unclear to what extent the precision or predictive value of a radiomic signature will be influenced by segmentation method, with variable results reported 37, 38, 39.

Another technical factor that can have a substantial effect on the calculation of texture features is the requirement for binning or quantization of the PET data, such that the original voxel intensity values are downsampled into a variable number of bins 17, 39, 40, 41. Bins are commonly equally divided into a set number (eg, 64 or 128), or the data can be placed into a variable number of bins of a fixed width (eg, 1 SUV unit). The latter method has been reported to correlate better with visual perception (42). Although no consensus has been reached for standardization for quantization in PET, using 64 equally divided bins has been a common approach (43). However, going forward, this is an important factor that requires standardization, given the large effect it can have on the measurement of texture features.

PET has relatively large voxels compared with MRI and CT, and the ability to accurately measure heterogeneity features without bias or dependence on volume is therefore more challenging. Using probability theory, Brooks et al. (25) calculated that a volume of 45 cm^3^ is required to adequately sample the tumor without significant bias on ^18^F-FDG PET images of cervical cancer from a scanner with 0.4 × 0.4 × 0.4 cm^3^ voxels. They reported that second-order entropy is dependent on the tumor volume but is 5 times more sensitive to changes in volume below the 45 cm^3^ threshold than above it (25). Another study reported that several texture features are highly correlated with the volume, that the correlation varies among different features, and the level of correlation significantly decreases with larger volume tumors (26). For example, second-order entropy showed high correlation in volumes of <10 cm^3^ but much less at volumes >10 cm^3^, suggesting a much lower minimum volume than 45 cm^3^ might be applicable. Rather than texture features being a surrogate for volume, that study showed in a subgroup of patients with non–small cell lung cancer (NSCLC) that heterogeneity and volume were independent prognostic factors and therefore complementary, especially in tumors >10 cm^3^ (26).

Although correlation between texture parameters and volume has been recognized, correlation between other radiomic parameters, including standard metrics such as SUV, is also common (41). This might either be due to similarities in the mathematical algorithm used to derive the features, leading to redundancy, or because an underlying biological process exists that affects more than 1 feature that might then be complementary to each other. It has been suggested that correlations between SUV and texture features can be minimized by using ≥32 bins (41). However, in addition to reporting correlations between texture features and standard metrics (eg, SUV or MV), using multivariable analyses or more robust statistical approaches with machine-learning techniques might be required to better understand or offset the effects of parameter correlation and redundancy 17, 44, 45.

An important factor in the decision of which texture features to use in a study in which serial measurements are required will depend on the repeatability or test-retest performance. Several parameters derived from ^18^F-FDG PET have been reported to be as repeatable as SUV (mean percentage difference ∼5%), including several local (eg, second-order entropy) and regional heterogeneity parameters describing variations in intensity and size of regions of homogeneous activity in 1 study using esophageal cancer ^18^F-FDG PET data (43). A further study reported 63 of 105 radiomic features (intensity, shape, and texture) with an intraclass correlation >0.9 in NSCLC data (46). Few parameters are robust to all the technical factors described, in particular, quantization and reconstruction algorithm, with lesser or variable, but measurable, effects from segmentation and smoothing 34, 38, 39, 40, 43, 47, 48.

Reaching consensus or being able to provide recommendations or guidelines from the existing radiomic and texture analysis data is problematic. Few systematic reviews or meta-analyses have been performed, resulting in difficulties with the adoption of radiomics into multicenter clinical trials and clinical practice. This is partly owing to the methodologic heterogeneity between reported studies and/or the lack of sufficient detail to replicate an analysis. Also, relatively few prospective studies with large numbers of patients that include training and (preferably external) validation cohorts to limit overfitting for testing specific image features or radiomic signatures have been reported. One review reported only 13% of studies (mainly MRI) adopted a prospective design, and 63% described training sets or cross-validation (21).

Other weaknesses in some of the data relate to the statistical analysis 21, 49. For example, when large numbers of image features are tested, a correction for multiple testing should normally be considered to avoid false-positive associations resulting from chance (eg, Holm-Bonferroni [50] or Benjamini-Hochberg [51] corrections), unless the study was purely observational or hypothesis generating. When this correction has been applied retrospectively in a systematic review, a large number of features in several studies were no longer statistically significant; this occurred in up to 45% of reviewed papers (21). Another potential factor that might increase the type 1 error rates unless a correction is applied is the use of optimal cutoffs to divide the data into high- and low-risk groups that maximize the statistical significance (48).

## Radiomics and Texture Analysis in Radiation Therapy

Interest is increasing in the use of radiomics in the radiation therapy community such that the term “radio-oncomics” has been proposed. Although most of the reported data have been on CT and MRI, potential applications exist for ^18^F-FDG PET/CT in this field (52).

Testing the hypothesis that tumors and normal tissues will show different textures, a pilot study aimed to identify the texture features from ^18^F-FDG PET/CT scans of head and neck squamous cell cancer that could improve the differentiation between tumor and normal tissues to optimize radiation therapy planning (53). Twenty-seven first-, second-, and high-order features and structural features were extracted from the PET and CT images, and K nearest neighbors and decision tree-based nearest neighbor classifiers were used. It was shown that PET and CT coarseness, contrast, and busyness had high discriminatory ability and that abnormal tissue was less uniform on PET but more uniform on CT. However, combining PET and CT features gave the best tissue characterization. Taking this further to a voxel-based approach, the same group tested an automated segmentation method using texture analysis from ^18^F-FDG PET/CT in head and neck cancer and showed high concordance with radiation oncologist delineations (54). Their findings argue that the method has the potential to reduce interobserver variability and improve treatment planning accuracy (54).

More commonly, texture analysis has been studied to evaluate the ability to predict or measure the treatment response. Heterogeneity on ^18^F-FDG PET usually infers a poor prognosis and decreases with successful treatment 55, 56, 57.

In patients with NSCLC receiving either conventional radiation therapy or stereotactic body radiation therapy (SBRT), a number of first-order features, calculated from the intensity-volume histogram of ^18^F-FDG PET/CT, were able to predict for local and locoregional control with a model of combined PET and CT features providing better predictors (58). High-order features, in particular, coarseness, from ^18^F-FDG PET have also shown predictive and prognostic capability in NSCLC patients who underwent chemoradiation therapy (57). A retrospective study of 26 patients with stage 1 NSCLC tested standard metrics and texture parameters in pretherapy (SBRT) ^18^F-FDG PET/CT scans for the prediction of local control, progression-free, and overall survival (59). Although most features showed good interobserver reproducibility, only the texture feature of high-intensity large area emphasis predicted for local control (*P* = .03) and SUVmax for progression-free survival (*P* = .03), with no PET parameters found for overall survival on univariate analysis. No correction was performed for multiple testing. A larger study of 63 patients with NSCLC who underwent SBRT tested standard metrics and 13 texture features in ^18^F-FDG PET/CT for disease-specific and overall survival (60). On multivariate analysis, only dissimilarity, a second-order feature derived from co-occurrence matrices, was associated with disease-specific and disease-free survival (hazard ratio 0.822, *P* = .037; hazard ratio 0.834, *P* < .01, respectively).

A number of studies using texture analysis to predict the response to chemoradiation therapy in esophageal cancer have been reported 55, 61, 62, 63. Tixier et al. (55) tested a range of first-, second-, and high-order features from ^18^F-FDG PET in a cohort of 41 patients who underwent definitive chemoradiation therapy for prediction of an eventual response measured using CT Response Evaluation Criteria In Solid Tumors on diagnostic CT scans. Although no global features were predictive, several local second-order features (eg, entropy; *P* = .0006) and regional high-order features (eg, coarseness; *P* = .0002) showed significant differences between responders and nonresponders (55). In a different study comparing pre- and post-therapy ^18^F-FDG PET/CT indeces with the pathologic tumor response for the prediction of response to neoadjuvant chemoradiation therapy, it was found that a decline in the mean SUV, pretherapy skewness, and post-therapy homogeneity were predictive (61). The same group then reported a support vector machine model that combined conventional PET parameters with heterogeneity measures and clinical parameters with perfect accuracy (area under the curve [AUC] 1.0) and no misclassifications (62). A similar study used least absolute shrinkage and selection operator regularization and logistic regression analysis to construct a model of histologic features, T stage, PET-derived long run low gray level emphasis, and CT-derived run percentage from a volume of interest incorporating the gross tumor volume from a rigidly coregistered radiation therapy planning scan to predict the pathologic response (AUC 0.78; after internal validation, AUC 0.74), which performed better than the SUVmax (AUC 0.58 and 0.54, respectively) (63).

The potential for PET or PET/CT radiomics has also been studied for the prediction of radiation toxicity. Reported data suggest that combining CT and ^18^F-FDG PET features from the lung might be able to predict for radiation pneumonitis in patients receiving radiation therapy for esophageal cancer (64) or that second-order features from ^18^F-FDG PET might predict radiation lung injury after SBRT for stage 1 NSCLC (65). Similarly, high levels of parotid ^18^F-FDG uptake and the texture feature of long run high gray level emphasis in patients with head and neck cancer, when added to a reference model (radiation dose and baseline xerostomia score), improved the prediction of radiation-induced xerostomia (66).

## Conclusions

Many challenges remain in the field of radiomics, not least, the need for consensus, reproducibility, standardization, and prospective validation in clinical trials 17, 67. Although PET has the advantage of being able to sensitively interrogate specific and varied abnormalities in tumor biology, its poorer resolution and variable noise pose additional technical limitations. Nevertheless, ^18^F-FDG PET/CT radiomics, frequently performed as texture analysis, have shown early promise in moving toward a personalized approach to radiation therapy and other oncologic therapy. Moving forward, it would seem likely that artificial intelligence and machine-learning methods will play a larger part in strengthening radiomic research and accelerating clinical translation.
